# ACTIVITY ENGAGEMENT FOR OLDER ADULTS WITH COGNITIVE IMPAIRMENT: UNDERSTANDING NEEDS AND SUPPORTS

**DOI:** 10.1093/geroni/igae098.4108

**Published:** 2024-12-31

**Authors:** Elizabeth Lydon, Carlyn Ellison Keeler, Raksha Mudar, Sara Czaja, Walter Boot, Neil Charness, Wendy Rogers

**Affiliations:** Miami University, Cincinnati, Ohio, United States; University of Illinois, Champaign, Illinois, United States; University of Illinois, Champaign, Illinois, United States; Weill Cornell Medicine/Center on Aging and Behavioral Research, New York, New York, United States; Weill Cornell Medicine, New York, New York, United States; Florida State University, Tallahassee, Florida, United States; University of Illinois, Champaign, Illinois, United States

## Abstract

Understanding the daily challenges experienced by older adults living with cognitive impairment is essential for developing strategies to support their health and quality of life. These impairments involve cognitive changes that can pose barriers to daily activities, especially complex tasks such as managing medications. However, the specific difficulties faced by this population are not well understood. The Everyday Needs Assessment for Cognitive Tasks (ENACT) Study examines these challenges in community-dwelling older adults with probable cognitive impairment determined by reporting recent changes in their cognition and scoring as mildly impaired on global cognitive screening. Participants were aged 60 and older (N = 91). We used a mixed methods needs assessment approach to identify difficulties across five activity domains: Health, Social Engagement, Transportation, Domestic Life, and Leisure and Recreation. Participants rated the level of difficulty they experienced with activities within these domains using a Likert scale and then participated in in-depth interviews to explore the underlying causes, focusing on memory, planning, organizing, and communication challenges. We examined participants’ response strategies to challenges, such as using visual reminders, technology, personal habits, and seeking assistance from others. Difficulties related to memory and executive functioning were key contributors to the participants’ challenges. Details from the interview data provide insights about the context of the challenges. Their most common response strategy was utilizing support from others. Interventions and technology solutions designed to support this population should prioritize features that aid memory recall and task organization and facilitate functional independence.

